# High Uterosacral Ligaments Suspension for Post-Hysterectomy Vaginal Vault Prolapse Repair

**DOI:** 10.3390/medicina60020320

**Published:** 2024-02-13

**Authors:** Marta Barba, Alice Cola, Tomaso Melocchi, Desirèe De Vicari, Clarissa Costa, Silvia Volontè, Lucia Sandullo, Matteo Frigerio

**Affiliations:** 1 Department of Gynecology, IRCCS San Gerardo dei Tintori, University of Milano-Bicocca, 20900 Monza, Italy; m.barba8792@gmail.com (M.B.); alice.cola1@gmail.com (A.C.); t.melocchi@campus.unimib.it (T.M.); desireedevicari@gmail.com (D.D.V.); c.costa14@campus.unimib.it (C.C.); s.volonte6@campus.unimib.it (S.V.); 2 Department of Gynecology, Università della Campania Luigi Vanvitelli, 81100 Caserta, Italy; lucia.sandullo@unicampania.it

**Keywords:** post-hysterectomy vault prolapse, native-tissue repair, high uterosacral ligaments suspension, transvaginal repair, pelvic organ prolapse

## Abstract

*Background and Objectives*: Uterosacral ligaments (USLs) suspension is a well-studied, safe, and long-lasting technique for central compartment correction. Preliminary clinical experiences showed encouraging data for this technique, also for post-hysterectomy vaginal vault prolapse surgical treatment. However, up-to-date evidence for post-hysterectomy vaginal vault prolapse repair through high uterosacral ligaments suspension is limited. Consequently, with this study, we aimed to assess the efficiency, complications frequency, and functional results of native-tissue repair through USLs in vaginal vault prolapse. *Materials and Methods*: This was a retrospective study. Women with symptomatic vaginal vault prolapse (≥stage 2) who underwent surgery with transvaginal native-tissue repair by high uterosacral ligaments were included. Patient characteristics, preoperative assessment, operative data, postoperative follow-up visits, and re-interventions were collected from the hospital’s record files. High uterosacral ligament suspension was performed according to the technique previously described by Shull. A transverse apical colpotomy at the level of the post-hysterectomy scar was performed in order to enter the peritoneal cavity. USLs were identified and transfixed from ventral to dorsal with three absorbable sutures. Sutures were then passed through the vaginal apex and tightened to close the transverse colpotomy and suspend the vaginal cuff. At the end of the surgical time, a diagnostic cystoscopy was performed in order to evaluate ureteral bilateral patency. Using the POP-Q classification system, we considered an objective recurrence as the descensus of at least one compartment ≥ II stage, or the need for a subsequent surgery for POP. The complaint of bulging symptoms was considered the item to define a subjective recurrence. We employed PGI-I scores to assess patients’ satisfaction. *Results*: Forty-seven consecutive patients corresponding to the given period were analyzed. No intraoperative complications were observed. We observed one postoperative hematoma that required surgical evacuation. Thirty-three patients completed a minimum of one-year follow-up (mean follow-up 21.7 ± 14.6 months). Objective cure rate was observed in 25 patients (75.8%). No patients required reintervention. The most frequent site of recurrence was the anterior compartment (21.2%), while apical compartment prolapse relapsed only in 6% of patients. An improvement in all POP-Q parameters was recorded except TVL which resulted in a mean 0.5 cm shorter. Subjective recurrence was referred by 4 (12.1%) patients. The mean satisfaction assessed by PGI-I score was 1.6 ± 0.8. *Conclusion*: This analysis demonstrated that native-tissue repair through high USL suspension is an effective and safe procedure for the treatment of post-hysterectomy vaginal vault prolapse. Objective, subjective, functional, and quality of life outcomes were satisfactory, with minimal complications.

## 1. Introduction

Hysterectomy is one of the most frequent gynecological surgical procedures, performed both for obstetric, benign, or malignant indications. Over time, the progressive passage to minimally invasive surgery asks the question of in which manner the pelvic floor may be influenced by surgery. Hysterectomy represents a risk factor for vaginal vault prolapse (VVP), which can require a subsequent surgical repair; moreover, the overall incidence of pelvic organ prolapse (POP) is rising due to the aging of the population. Other risk factors for post-hysterectomy vaginal prolapse are parity, obesity, connective tissue characteristics, age, and chronic lung conditions [1,2,3,4,5,6]. Indeed, despite any indication for which hysterectomy is performed being related to an increased risk of urogenital prolapse, additional evidence suggests that this risk is increased if hysterectomy is performed for prolapse. This is highlighted in two case-control studies which have demonstrated that previous pelvic floor surgery is the greatest risk factor for developing VVP and that risk of prolapse repair is 4.7 times higher if uterine prolapse was the prior reason for vaginal hysterectomy. A subsequent nationwide cohort study confirmed these data: having a vaginal hysterectomy for prolapse was demonstrated to be a greater risk factor compared to other indications for hysterectomy. Evidence suggests that, additionally, other different types of pelvic surgery may increase the risk of postoperative prolapse, including colposuspension, previous pelvic floor surgery, and rectopexy [7]. Overall, among people treated by vaginal hysterectomy for prolapse, long-term prevalence of VVP has been estimated in 23% [8]. The longer the time after hysterectomy, the higher the risk of VVP. Due to improved life expectancy, the increasing age of the female population will lead to an extra demand for future prolapse surgery.

The symptoms of VVP are usually pelvic pressure, bulging and dropping sensation of vagina walls, or backache. Moreover, other symptoms can be present, related to bladder or bowel dysfunctions due to the dislocation of these structures. For this reason, many surgical operations for VVP try to go in the direction of re-establishment of the normal anatomy of the vagina, which is strictly linked to the normal function of these districts [9,10,11,12].

Several techniques have been described for VVP surgery, including vaginal, laparoscopic, and abdominal approaches. Apical support represents the main-stay for a successful repair [13]. The abdominal approach is usually performed in the form of sacral colpopexy, with a success rate in maintaining apical support from 78–100% during a follow-up period of 6 months to 3 years. This technique is generally considered the gold standard for post-hysterectomy vaginal vault prolapse due to excellent anatomical outcomes. However, the limitation of abdominal approaches is the need for a wide dissection, which may lead to significant venous bleeding from presacral veins. Moreover, it is well established that abdominal surgery has higher rates of morbidity and longer convalescent times than vaginal or laparoscopic surgery [14,15]. On the other hand, minimally invasive surgery used in the field of reconstructive gynecologic procedures has many advantages: the rates of postoperative infections and pain after surgery are less with a minimally invasive approach, and so the time needed to return to work or do daily activities is faster than after open surgery. In the end, this approach can guarantee an improvement also from the point of view of cosmetic results [16,17]. Thanks to the spread of mini-invasive surgery, abdominal sacral colpopexy has also found indication to be performed both laparoscopically and robotically. The benefits of mini-invasive approaches are that an optimal visualization of pelvic anatomy and presacral space is guaranteed. Both laparoscopic and robotic sacral colpopexy routes have been described in the literature and showed good success rates [18]. Moreover, the procedures based upon the use of uterosacral ligaments for suspension can be performed using laparoscopic or robotic techniques [19].

The original type of minimally invasive surgery is the vaginal approach. Women undergoing vaginal procedures do not need external incisions and pain is minimized due to the fact that there is no break of the abdominal skin, fascia, and muscles [20,21]. After the introduction of synthetic mesh for the use in vaginal POP surgery, with the aim of more durable and effective treatment results, most of this kind of product have now been removed from the market, because they unfortunately resulted in high numbers of unexpected adverse events [22]. The renewed interest that characterized recent times is about the possibility of mesh-free procedures to be utilized thanks to their inferior costs and the obvious absence of graft-related complications. The key point underlined by detractors of these techniques is that native-tissue procedures could be associated with a higher risk of recurrence in the long term. Main risk factors proposed are age and other characteristics of the population, such as obstetric history, and specific items such as the prolapse stage, histologic findings, and supplementary surgical procedures performed (such as anterior or posterior repair) [23,24].

Vaginal native-tissue techniques comprehend sacrospinous ligament (SSL) fixation, iliococcygeus fascia (ICF) fixation, levator myorrhaphy (LM), and uterosacral ligament (USL) suspension. SSL fixation is one of the most popular transvaginal techniques for VVP repair. First described by Sederl in 1958, this technique implies the suspension of vaginal apex to sacrospinous ligaments either unilaterally or bilaterally [25]. The surgical repair of apical prolapse can be achieved using the sacrospinous ligament (SSL), especially if minimal invasiveness and effectiveness are desired. Indeed, compared to abdominal sacrocolpopexy and vaginal hysterectomy with uterosacral ligament suspension, this approach seems to be less extensive and less expensive both from the point of view of the operation time and the recovery of the patient, as the time before a return to daily routine activities is shorter. Moreover, this technique seems to have fewer complications than abdominal sacrocolpopexy and vaginal hysterectomy with uterosacral ligament suspension. Currently, there are different vaginal surgical approaches described to access the SSL to enable the suspension of the vaginal cuff. The classical Amreich–Richter technique involves the direct visualization of the SSL [26]. This technique requires a posterior colpotomy, and then that the rectal stalk is bluntly penetrated and prepared up to the ischial spine, just for obtaining a good exposition of the sacrospinous ligament to correctly place 2–3 sutures. So, the vaginal vault is included subepithelially and the sutures are tied on the sacrospinous ligament. The need for an optimal visualization of the SSL sometimes can be technically challenging due to the deep vaginal dissection which is required [27]. This critical issue can be overcome by the so called “blunt and blind” dissection method, which targets the midpoint of the SSL. This technique seems to be useful in order to reduce the risk of infection and bleeding associated with the previously described approach. The placed sutures can be used for direct attachment of the vaginal vault to the SSL, as well as indirect fixation with the use of mesh implants. The closed SSL fixation represents a more recent development in which the surgeon palpates the SSL transvaginally and anchors the suture through the vagina without surgical dissection of the pararectal space down to the ligament. This suture is then used for vaginal vault suspension. Hemorrhage is rare due to the fact that wide dissection is avoided, and mesh complications are omitted thanks to the employ of sutures only [28]. Again, sacrospinous ligament suspension, although it has been demonstrated to be successful in suspending the prolapsed vaginal vault, has been unfortunately associated with postoperative rates of cystocele formation in 18–92% [29,30].

Iliococcygeus fascia (ICF) fixation, after the first description by Inmon in 1963, has been revised by Shull in 1993 [31,32]. In this technique, the first surgical step is aimed at the preparation of the pararectal space; then, the vaginal apex is attached to the ICF and muscle, just below the level of the ischial spine. It is easy to understand the importance when performing this kind of surgery of being aware of obtaining an adequate pararectal space dissection to have an ideal visualization of the iliococcygeus muscle: to minimize the risk of rectal injury. Taking into consideration these aspects, ICF fixation is a simple surgical technique and is associated with minimal morbidity. Nevertheless, it is not commonly performed, probably due to the not so irrelevant tax of mild complications, such as temporary urinary retention, urinary tract infections, and vaginal granuloma [33]. However, iliococcygeus fascia fixation may result in vaginal foreshortening because the point of apical fixation is distal to the ischial spines [34,35,36].

Levator myorrhaphy (LM) is a procedure first presented by Francis and Jeffcoate in 1961 [37]. This procedure is characterized by the wide midline plication of the levator ani muscles to which the vaginal cuff is attached. Few data are available for this technique, because its use is quite rare, since it is considered a constricting technique. Overall, the procedure is generally considered with low morbidity, but some relevant complications have been described, such as rectal laceration, bladder tear, vaginal cuff abscess, pararectal hematoma, and buttock pain [38,39].

Uterosacral ligament (USL) suspension is a well-established, safe, and durable technique for central compartment correction [40,41,42]. Clinical experiences showed encouraging data also for post-hysterectomy vaginal vault prolapse surgical treatment. Moreover, the uterosacral vault suspension is usually able to maintain the right orientation of the vaginal axis, so potentially preventing the recurrence rate of prolapse in other vaginal compartments, especially the anterior one.

In our institution, we routinely perform native-tissue repair through high uterosacral ligaments suspension (USLs), for uterovaginal, uterine-sparing, and vaginal vault prolapse. However, up-to-date evidence for post-hysterectomy vaginal vault prolapse repair through high uterosacral ligaments suspension is limited. Consequently, with this study, we aimed to evaluate the effectiveness, complications rate, and functional results of native-tissue repair through USLs in vaginal vault prolapse [43].

## 2. Materials and Methods

This was a retrospective study. Approval from the local Ethics Committee was obtained before the start of the study (protocol code: SH-MCC). Patients with symptomatic vaginal vault prolapse (≥stage 2) who had been treated with transvaginal native-tissue repair by high uterosacral ligaments suspension between 2017 and 2022 were included. Patient characteristics, preoperative assessment, operative data, postoperative follow-up visits, and re-interventions were collected from the hospital’s record files.

Preoperative evaluation included a medical interview and clinical examination. Obstetric history, the presence of genito-urinary symptoms, lower urinary tract symptoms, and sexual and bowel disorders were evaluated during clinical interview. The Pelvic Organ Prolapse Quantification system (POP-Q) was utilized to stage vaginal prolapse [44].

**Figure 1:**
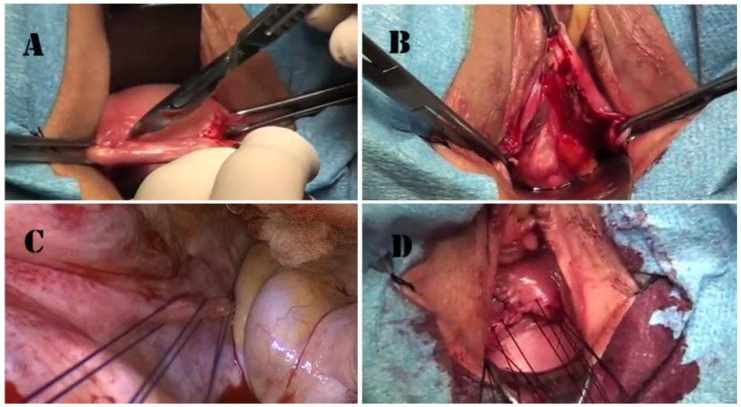
Surgical technique. (**A**) Apical colpotomy at the level of the scar of the previous hysterectomy; (**B**) Douglas pouch opening; (**C**) triple high uterosacral ligament transfixion (image was acquired for didactical purpose through a laparoscopic camera by vaginal route to better demonstrate the USL transfixion); (**D**) aspect after the passage of the suspending sutures through the vaginal apex, just before sutures tighten.

**Figure 2:**
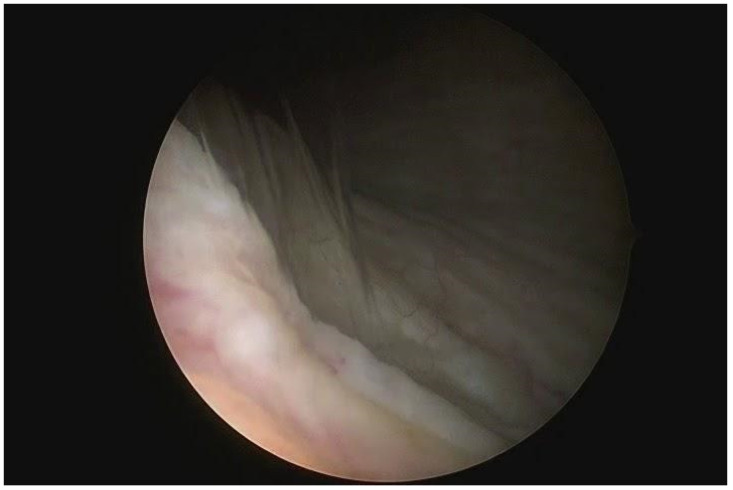
Diagnostic cystoscopy to evaluate ureteral patency. Demonstration of the left ureteral jet.

High uterosacral ligament suspension was performed according to the technique previously described by Shull [45]. A transverse apical colpotomy at the level of the post-hysterectomy scar was performed in order to enter the peritoneal cavity (Figure 1A,B). USLs were identified and transfixed from ventral to dorsal with three absorbable sutures (Figure 1C). The lowest suture was placed at the level of the ischial spine, and the followings were placed 1 cm (0.4 in) and 2 cm (0.8 in) above the first one. Sutures were then passed anteriorly through the peritoneum and anterior vaginal wall, and posteriorly through the peritoneum and posterior vaginal wall. Distal USLs sutures were passed laterally, the proximal ones medially, and the intermediate ones between the previous two (Figure 1D). All sutures were tightened in order to close the transverse colpotomy and suspend the vaginal cuff. Diagnostic cystoscopy was performed at the end of the surgical time in order to assess ureteral bilateral patency (Figure 2) [46,47].

Postoperative follow-up visits were performed 1, 6 and 12 months postoperatively and then annually. Objective recurrence was defined as the descent of at least one compartment ≥ II stage according to the POP-Q system or the need for reoperation for POP. Subjective recurrence was defined as the presence of bulging symptoms. Clinical interview assessed the presence of genito-urinary symptoms, lower urinary tract symptoms, sexual and bowel disorders. The patients’ satisfaction was evaluated with PGI-I scores [48,49]. This is a seven-point scale quality of life (QoL) questionnaire evaluating patients’ satisfaction with a range of responses from 1, “very much improved” to 7, “very much worse”. For patients lost at follow-up, a phone call was performed, and if answered they were asked to perform an outpatient visit.

Data were entered into the database by one author and double-checked by one other author. Descriptive statistics were calculated as absolute numbers with percentages for categorical variables and as mean (standard deviation) for continuous ones. Statistical analysis was performed using JMP software version 9.0. Differences were tested with the paired test for continuous data and with the Fisher’s test for non-continuous data. A *p* < 0.05 was considered significant.

## 3. Results

According to our inclusion criteria, 47 consecutive patients corresponding to the given period were analyzed. Population characteristics are summarized in Table 1. Operative data are shown in Table 2. The apical suspension was achieved by USL suspension in all patients. Additional procedures were performed as follows: 70.2% anterior and 59.6% posterior repair, respectively. No intraoperative complications were observed. We observed one postoperative hematoma that required surgical evacuation.

Thirty-three patients completed a minimum of one-year follow-up (loss at follow-up 29.8%). The mean follow-up was 21.7 ± 14.6 months. Objective outcomes are shown in Table 3. Objective cure rate was observed in 25 patients (75.8%). Notably, half of them were asymptomatic, and the remaining only had mild symptoms, and no patients required reintervention. The most frequent site of recurrence was the anterior compartment (21.2%), while apical compartment prolapse relapsed only in 6% of patients. No differences in terms of recurrence rates were observed depending on previous hysterectomy indications (prolapse vs other benign indications). An improvement in all POP-Q parameters was recorded except TVL which resulted in a mean 0.5 cm shorter (Table 4). Subjective and functional outcomes are shown in Table 5. Surgical repair resulted in a reduction of vaginal bulging and voiding symptoms. Stress incontinence, overactive bladder syndrome, and constipation rates were not modified following treatment. Similarly, sexual function was not affected by surgery. Subjective recurrence was referred by 4 (12.1%) patients. The mean satisfaction evaluated with the PGI-I score was 1.6 ± 0.8.

## 4. Discussion

Several surgical techniques for vaginal vault prolapse repair are described in the literature, using different suspending structures based on the chosen technique. Among them, since the Food and Drug Administration alert related to surgical mesh use in urogynecological surgery, the transvaginal POP mesh repairs should be performed less and less, due to the not indifferent risk of mesh exposure and erosion. This consideration made necessary in-depth analysis about native-tissue procedures and suspending techniques to report and study long-term outcomes. Among the latter, uterosacral ligament suspension (USLs) is a well-established surgical option for primary prolapse repair as well as vaginal vault prolapse correction, even if few data are available in the literature compared to other techniques. Potential advantages include the preservation of the natural orientation of the vaginal axis, and the well-established efficacy and safety in previous studies for primary prolapse repair. The main pitfall is the risk of ureteral obstruction described for this procedure.

This study aimed to evaluate outcomes of transvaginal native-tissue repair through uterosacral ligaments suspension (USLs) for post-hysterectomy vaginal vault prolapse. Our purpose was to understand if there are significant limitations, as well as important advantages, to the utilization of this technique compared to the other surgical procedures described in the literature for this type of prolapse. We found that USL suspension gave safe and effective results in correcting post-hysterectomy vaginal vault prolapse, with objective and subjective cure rates, reoperations and perceived satisfaction comparable to those reported for different apical suspension techniques.

Historically, abdominal sacrocolpopexy (ASC) has been considered the gold standard for VVP repair. In a systematic review of 2016, Campbell et al. reported a mean duration of surgery for abdominal sacrocolpopexy (ASC) of 187 min and a mean intraoperative blood loss of 232 mL [43]. The same review reported a mean duration of surgery for abdominal sacrocolpopexy performed by laparoscopic route of 213 min and a mean intraoperative blood loss of 121 mL [50]. Compared to the results obtained in our study performing USLs, it is easy to understand how this approach seems to be significantly faster than ASC, both performed open and via laparoscopy, and with similar quantities of blood loss. On the other hand, even if ASC is considered in the literature as the gold standard for the treatment of vaginal vault prolapse due to better anatomical outcomes, in this study we found very good results also from this point of view, with an objective cure rate observed in 75.8% of patients, with no need of subsequent surgery. The most frequent site of recurrence was the anterior compartment (21.2%), while apical compartment prolapse relapsed only in 6% of patients. Moreover, an improvement in all POP-Q parameters was recorded. TVL resulted in a mean only 0.5 cm shorter, an outcome that readily explains how the suspension can be optimal with this technique, as it influences several clinical aspects such as the post-operative possibility of maintaining good sexual activity. So, we can affirm that USLs seems to be equally effective from the point of view of anatomical outcomes and surgical results.

Regarding native-tissue transvaginal repair or post-hysterectomy, many procedures are available, including sacrospinous ligament fixation, uterosacral ligaments suspension, iliococcygeus fascia fixation, and levator myorrhaphy. Among them, sacrospinous fixation is the one with most evidence. Specifically, this technique seems to have some advantages compared to sacropexy regarding the operation time and incidence rate of functional postoperative tools such as dyspareunia [51]. Another study compared outcomes of all transvaginal native-tissue procedures for post-hysterectomy vaginal vault prolapse repair and investigated differences [34]. Data showed that all native-tissue procedures have low complication rates and reassuring objective, subjective and functional outcomes, without any significant differences in terms of complications and outcomes, with the exception of vaginal profile which is narrower and shorter after levator myorrhaphy compared to other apical suspensions. The safety profile of this procedure was also found to be satisfactory, and we did not record any complications, except for a postoperative hematoma. In particular, no ureteral injuries occurred. This may be due to the routine use of intraoperative diagnostic cystoscopy, which allows intraoperative recognition and management of ureteral obstruction without postoperative sequelae.

To our knowledge, this is the first study focusing on outcomes after native-tissue repair through high uterosacral ligaments suspension for post-hysterectomy vaginal vault prolapse. Another strength involves the multimodal evaluation of surgical success. Limitations include the retrospective study design and the limited population, which, however, depends on the minor prevalence of post-hysterectomy vaginal vault prolapse compared to primary utero-vaginal prolapse. Another limitation may be related to the fact that dissatisfied patients with a recurrence can seek treatment at other hospitals, so creating a loss at follow-up; in this way, the failure rate could be underestimated. Despite this limitation, our loss rate was similar to those of previous works of this type.

## 5. Conclusions

This analysis demonstrated that native-tissue repair through high USL suspension is an effective and safe procedure for the treatment of post-hysterectomy vaginal vault prolapse. Objective, subjective, functional, and quality of life outcomes were satisfactory, with minimal complications. Consequently, this technique should be considered as an option to treat vaginal vault prolapse, especially in cases of old or frail patients, in which reduced comorbidities and avoidance of mesh-related complications are priorities. However, prospective data and larger and comparative studies (e.g., comparison with SSF) are necessary to confirm our findings.

## Figures and Tables

**Table 1:** Population baseline characteristics. Continuous data are reported as mean (SD). Non-continuous data are reported as absolute (relative) frequency.

Age (Years)	64.8 ± 9.6
Parity (n)	2.0 ± 0.8
Menopausal status	45 (95.7%)
BMI (Kg/m^2^)	27.0 ± 6.1
ASA status 1	4 (8.5%)
ASA status 2	35 (74.5%)
ASA status 3	8 (17.0%)
Indication for previous hysterectomy	27 (57.4%) Uterovaginal prolapse
	20 (42.6%) Other benign indications

**Table 2:** Operative data. Continuous data are reported as mean (SD). Non-continuous data are reported as absolute (relative) frequency. * Hematoma requiring re-surgery.

High Uterosacral Ligaments Suspension	47 (100%)
Anterior repair	33 (70.2%)
Posterior repair	28 (59.6%)
Blood loss (mL)	184 ± 126
Operative time (min)	82 ± 26
Intraoperative complications	0
Postoperative complications	1 (2.1%) *

**Table 3:** Objective and subjective outcomes. Continuous data are reported as mean (SD). Non-continuous data are reported as absolute (relative) frequency. PGI-I: Patient’s Global Impression of improvement (Very much better = 1; Much better = 2; A little better = 3; No change = 4; A little worse = 5; Much worse = 6; Very much worse = 7).

Anatomic Recurrence	8 (24.2%)
- Anterior recurrence	7 (21.2%)
- Central recurrence	2 (6.0%)
- Posterior recurrence	1 (3.0%)
Reoperation	0 (0%)
Postoperative bulging symptoms	4 (12.1%)
PGI-I	1.6 ± 0.8

**Table 4:** POP-Q comparison. Continuous data are reported as mean (SD). Aa: fixed point, midline of anterior vaginal wall, located 3 cm proximal to external urethral meatus; Ba: moving point, most distal portion of any part of anterior vaginal wall; C: leading edge of vaginal cuff (post-hysterectomy); gh: genital hiatus; pb: perineal body; tvl: total vaginal length; Ap: fixed point, midline of posterior vaginal wall, located 3 cm proximal to hymen; Bp: moving point, most distal portion of any part of posterior vaginal wall.

	Preoperative	Postoperative	*p*-Value
Aa	1.1 ± 1.7	−1.8 ± 1.2	<0.001
Ba	1.3 ± 1.9	−1.8 ± 1.2	<0.001
C	1.2 ± 2.7	−5.3 ± 2.7	<0.001
gh	4.1 ± 0.7	3.6 ± 0.6	<0.001
pb	2.8 ± 0.5	3.1 ± 0.5	0.01
tvl	8.6 ± 1.0	8.1 ± 1.2	0.004
Ap	−1.7 ± 1.4	−2.7 ± 0.6	<0.001
Bp	−1.5 ± 1.6	−2.7 ± 0.6	<0.001

**Table 5:** Functional comparison. Non-continuous data are reported as absolute (relative) frequency.

	Preoperative	Postoperative	*p*-Value
Stress urinary incontinence	16 (34.0%)	6 (18.2%)	0.135
Voiding symptoms	29 (61.7%)	6 (18.2%)	<0.001
Overactive bladder syndrome	18 (38.3%)	10 (30.3%)	0.486
Constipation	13 (27.7%)	11 (33.3%)	0.626
Bulging symptoms	47 (100%)	4 (12.1%)	<0.001
Sexual activity	15 (31.9%)	10 (30.3%)	1

## Data Availability

The data presented in this study are available on request from the corresponding author.
